# Supplementary material for: Gluconeogenesis in the yolk syncytial layer‐like tissue of cloudy catshark (Scyliorhinus torazame)
Source: Physiol Rep. 2024 May 29;12(11):e16088. doi: 10.14814/phy2.16088 (PMC11136554; doi:10.14814/phy2.16088)
Supplement: Supplementary file 1 — Figure S1. [file PHY2-12-e16088-s001.pdf]

Supplementary Figure 1. Shimizu et al.

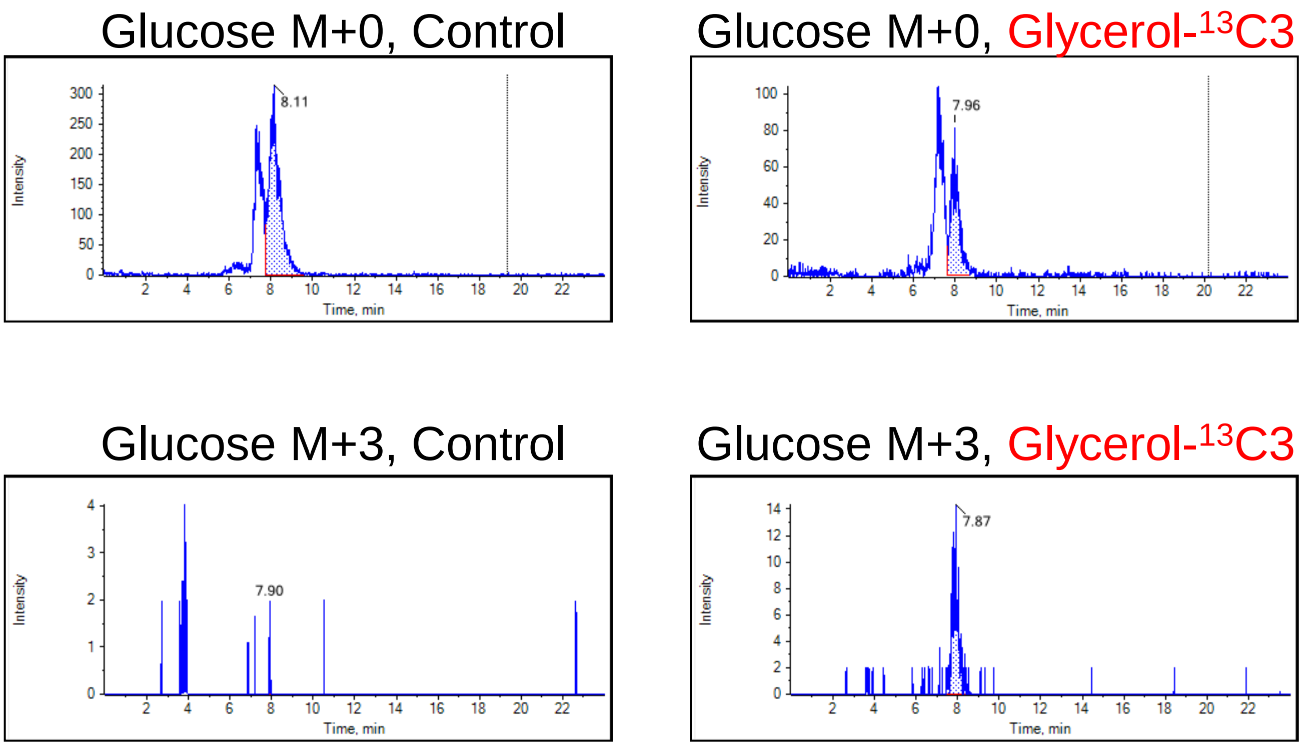

Fig. S1. The examples of chromatograms for glucose M+0 (top panels) and glucose M+3 (bottom panels). Left, control samples; right, samples incubated with glycerol-^{13}C3. For the representation of levels of glucose M+3, the value of (glucose M+3 area / glucose M+0 area)*100 was used. The peaks seen ~1 min earlier in retention time than glucose represent galactose, which can be distinguished by comparing with standard solutions.

Supplementary Figure 2. Shimizu et al.

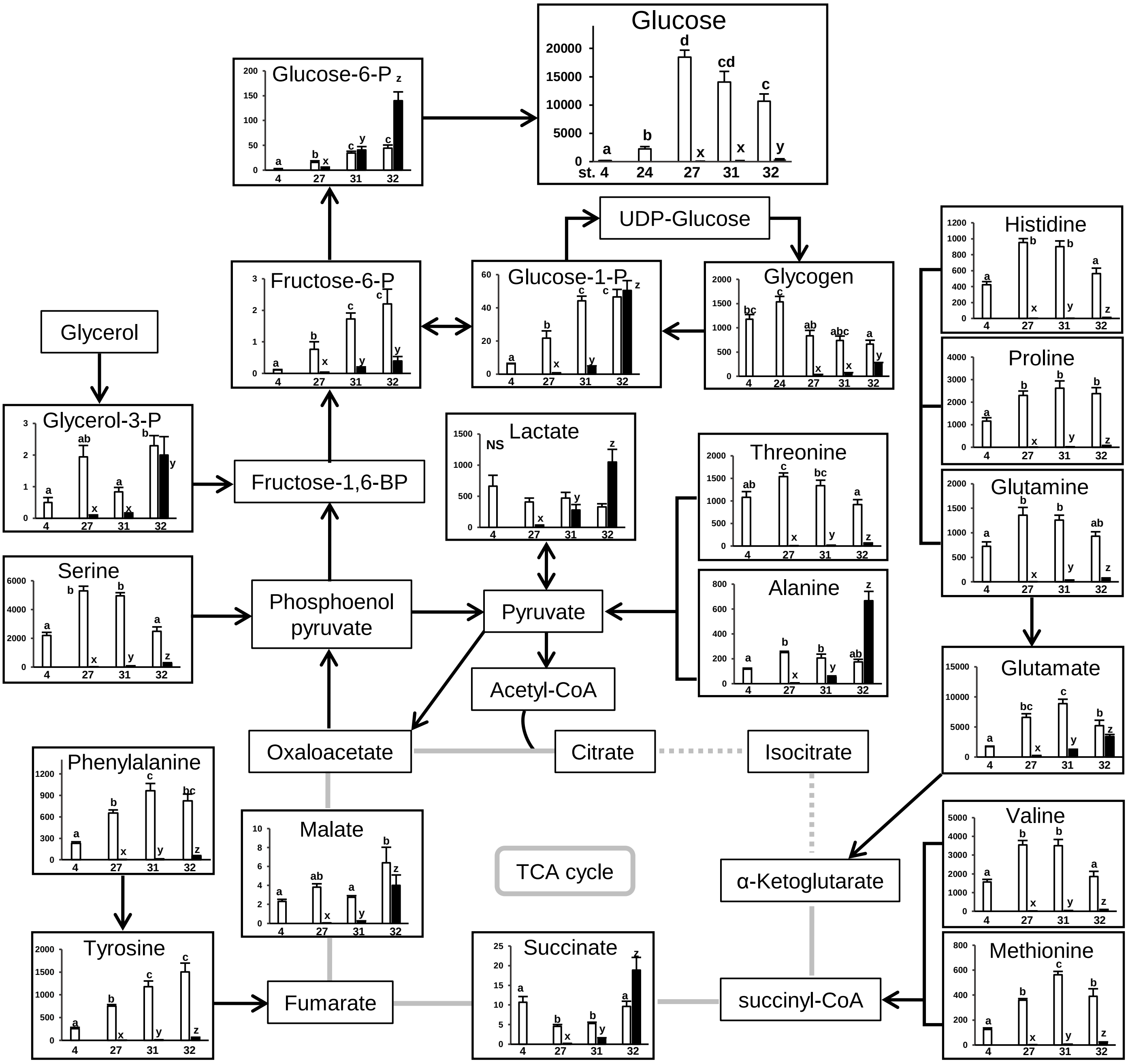

Fig. S2. The metabolic pathway map showing changes in each metabolite levels per individual yolk sac (open column) or embryo (filled column) during development. The horizontal axes represent developmental stages {stages 4, 24 (glucose and glycogen only), 27, 31, 32} and the vertical axes represent nmol / sample. Data are presented as mean ± standard error (N = 6). Different letters indicate significant differences ($P < 0.05$) between groups. Tests for significant differences were performed by one-way ANOVA and Tukey's *post-hoc* test separately for yolk sac or embryo samples after log transformation. Fructose-1,6BP, fructose 1,6-bisphosphate; -P, -phosphate.

Supplementary Figure 3. Shimizu et al.

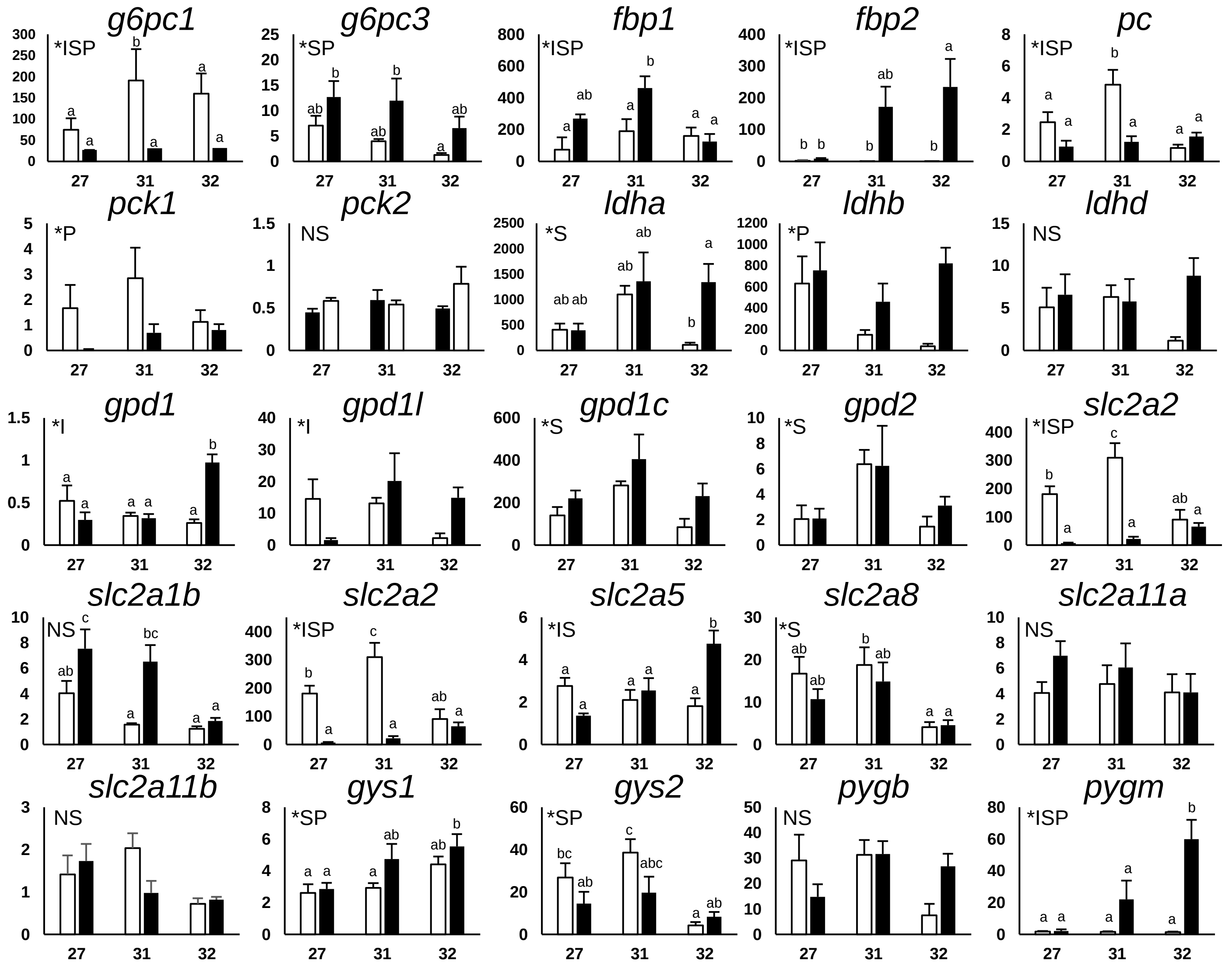

Fig. S3. Changes in expression levels of the selected genes during development.

The expression levels of gluconeogenesis-related genes and glucose transporter (GLUT) gene (*slc2a*) family were measured. *slc2a2* were measured. Horizontal and vertical axes indicate developmental stages and mRNA levels (x 10^9 copies / g RNA), respectively. Open and filled columns indicate the mRNA levels in the yolk sac membrane and embryos, respectively. Data are presented as mean ± standard error (N = 6), and different letters indicate significant differences ($P < 0.05$) between groups. Tests for significance were performed by two-way ANOVA and Tukey's *post-hoc* test. *I indicates a significant interaction between the two factors (developmental stage and site), and *S and *P indicate significant main effects of developmental stage and the position (yolk sac or embryo), respectively.

Supplementary Figure 4. Shimizu et al.

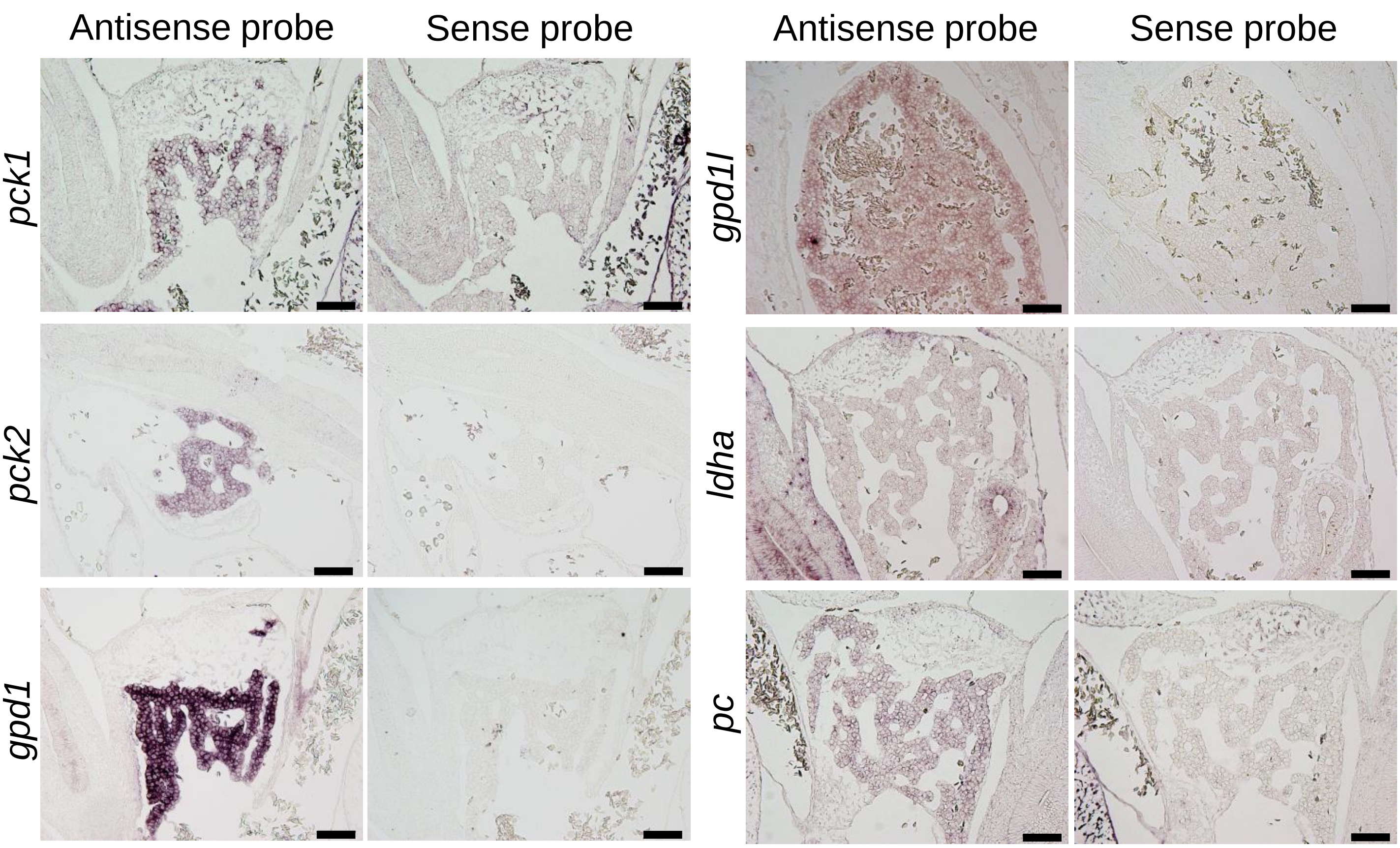

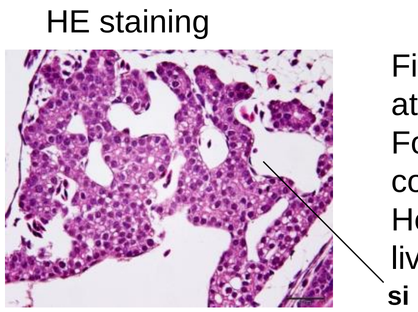

Fig. S4. *In situ* hybridization for *pck1*, *pck2*, *gpd1*, and *ldha*, in the liver at stage 27.
For all transcripts, sense probes (right) were used as negative controls for antisense probes (left), which showed true signals. Hematoxylin and eosin (HE) staining shows general morphology of liver in stage 27. si, sinusoids. Scale bars = 100 μm.

Supplementary Figure 5. Shimizu et al.

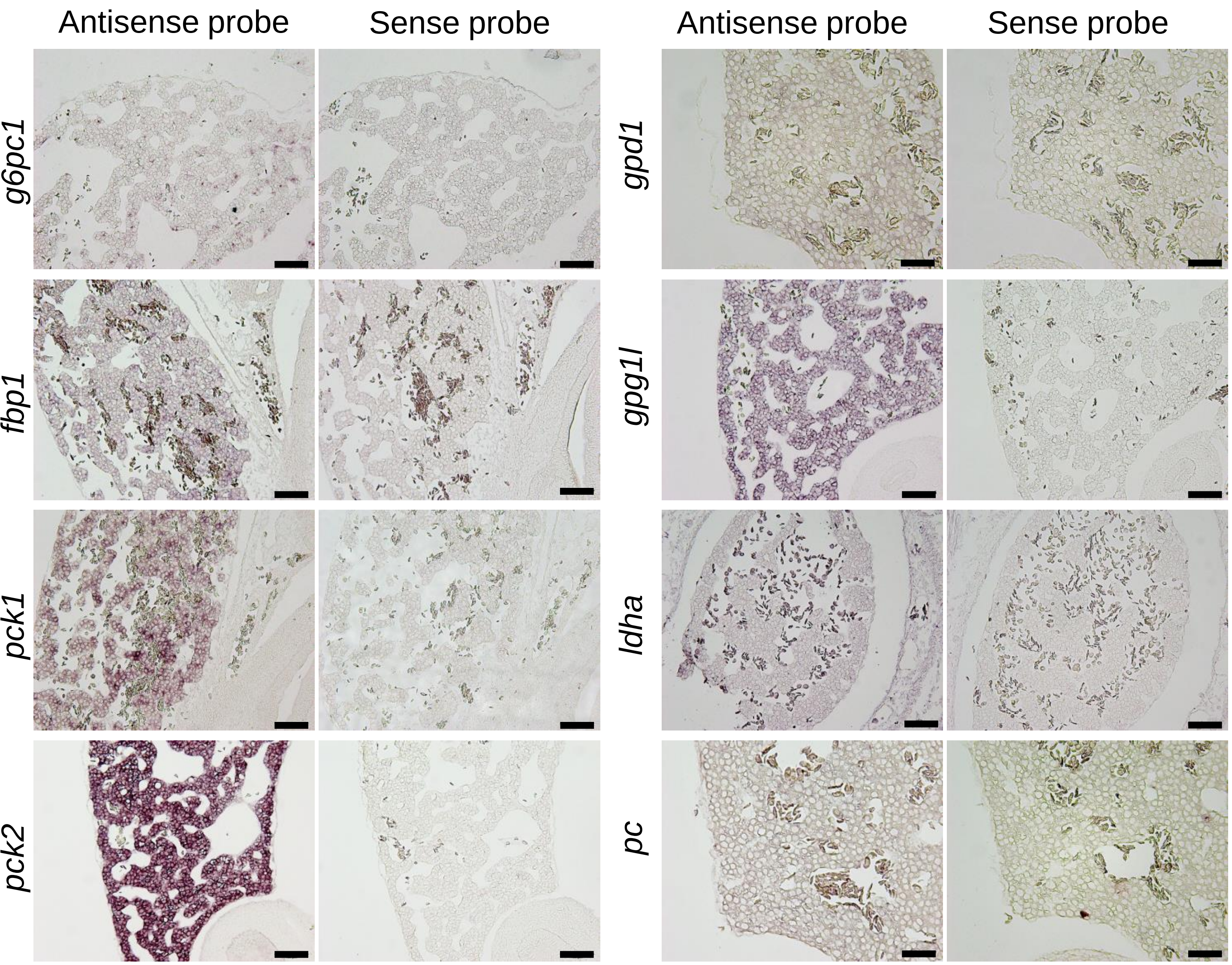

Fig. S5. *In situ* hybridization for *g6pc1*, *fbp1*, *pck1*, *pck2*, *gpd1*, *gpd1l*, *ldha* and *pc* in the liver at stage 31.
For all transcripts, sense probes (right) were used as negative controls for antisense probes (left), which showed true signals. Scale bars = 100 μm.

Supplementary Figure 6. Shimizu et al.

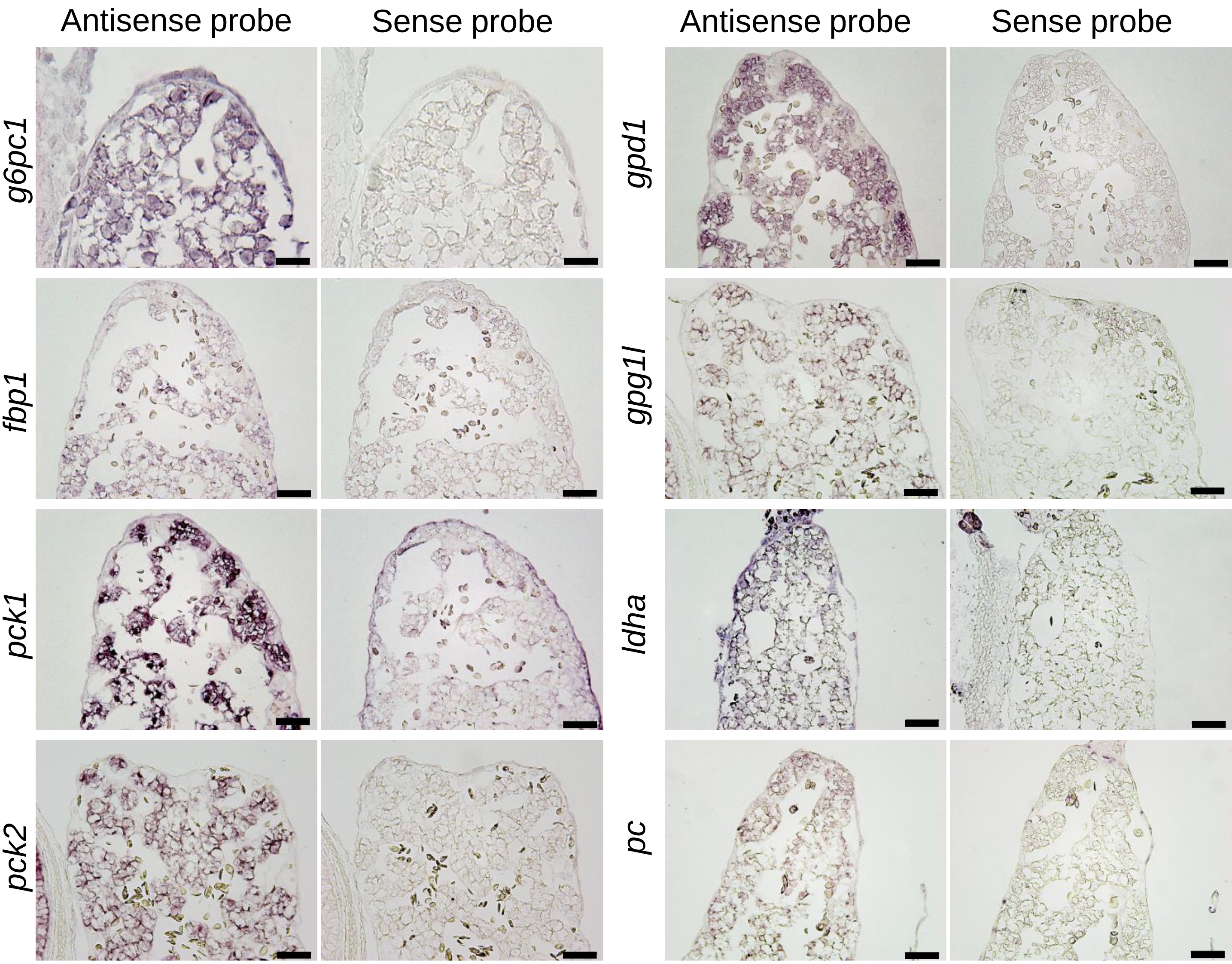

Fig. S6. *In situ* hybridization for *g6pc1*, *fbp1*, *pck1*, *pck2*, *gpd1*, *gpd1l*, *ldha* and *pc* in the liver at stage 32.
For all transcripts, sense probes (right) were used as negative controls for antisense probes (left), which showed true signals. Scale bars = 50 μm.
