# Supplementary material for: Gluconeogenesis in the yolk syncytial layer‐like tissue of cloudy catshark (Scyliorhinus torazame)
Source: Physiol Rep. 2024 May 29;12(11):e16088. doi: 10.14814/phy2.16088 (PMC11136554; doi:10.14814/phy2.16088)
Supplement: Supplementary file 2 — Table S1. [file PHY2-12-e16088-s002.docx]

| Supplementary table 1. The characteristics and weights of samples. | |  |
| --- | --- | --- |
| Stages | Characteristics | Wet weight in average ± standard error (g) |
| Stage 4 | immediately after spawning, characterized by appearance of “caudal crescent” | 1.811 ±0.117 |
| Stage 24 | the opening of first four pairs of pharyngeal clefts | 1.97 ± 0.070 |
| Stage 27 | appearance of gill filament buds, completion of vascularization of the yolk sac | e: 0.030 ± 0.003 |
|  |  | y: 1.857 ± 0.080 |
| Stage 31 | appearance of the rostrum | e: 0.133 ± 0.015 |
|  |  | y: 1.812 ± 0.101 |
| Stage 32 | (rostrum-body axis angle becomes less than 90°) | e: 0.673 ± 0.059 |
|  |  | y: 1.412 ± 0.125 |
| e, embryonic body; y, yolk sac | |  |
